# Voltammetric determination of hydrogen peroxide at decorated palladium nanoparticles/poly 1,5-diaminonaphthalene modified carbon-paste electrode

**DOI:** 10.1098/rsos.231894

**Published:** 2024-06-12

**Authors:** Ziad Khalifa, Mohamed Fathi Abo Oura, Abla Hathoot, Magdi Abdel Azzem

**Affiliations:** ^1^ Chemical Engineering Department, Faculty of Engineering, The British University in Egypt, El Sherouk City 11837, Egypt; ^2^ Electrochemistry Laboratory, Chemistry Department, Faculty of Science, Menoufia University 32512, Egypt

**Keywords:** hydrogen peroxide, modified electrodes, palladium nanoparticles, sensors, square wave voltammetry

## Abstract

In this work, palladium nanoparticles (PdNPs)/p1,5-DAN/ carbon paste electrode (CPE) and p1,5-DAN/CPE sensors have been developed for determination of hydrogen peroxide. Both sensors showed a highly sensitive and selective electrochemical behaviour, which were derived from a large specific area of poly 1,5 DAN and super excellent electroconductibility of PdNPs. PdNPs/p1,5-DAN/CPE exhibited excellent performance over p1,5-DAN/CPE. Thus, it was used for detecting hydrogen peroxide (H_2_O_2_) with linear ranges of 0.1 to 250 µM and 0.2 to 300 µM as well as detection limits (*S/N = 3*) of 1.0 and 5.0 nM for square wave voltammetry (SWV) and cyclic voltammetry (C.V) techniques, respectively. The modified CPE has good reproducibility, adequate catalytic activity, simple synthesis and stability of peak response during H_2_O_2_ oxidation on long run that exceeds many probes. Both reproducibility and stability for H_2_O_2_ detection are attributable to the PdNPs immobilized on the surface of p1,5-DAN/CPE. The modified CPE was used for determining H_2_O_2_ in real specimens with good stability, sensitivity, and reproducibility.

## 1. Introduction

Hydrogen peroxide (H_2_O_2_) is widely used in industrial and medical applications [1,2]. Biologically reactive oxygen species is produced from cell oxidases, which help the signal transduction of cells [3]. Excess use of H_2_O_2_ is associated with defects in cell growth, genetic carrier (DNA), and cytokines damage [4,5]. H_2_O_2_ can be determined by chemiluminescence [6], colorimetry [7], titrimetry [8], spectrophotometry [9] and fluorescence [10] techniques. However, these techniques are time-consuming, expensive, complex and suffer from interference with other analytes.

Electrochemical methods have important applications such as sample analysis as well as organic and inorganic synthesis [11]. These methods are attractive for biological and environmental analysis because they are cheap, simple, fast, sensitive and selective [12]. For example, electrochemical assays were used for H_2_O_2_ detection, with high sensitivity and selectivity [13]. H_2_O_2_ interferes with oxygen and its oxidation peak, which allows adequate detection rather than using the reduction peak [14]. The direct oxidation or reduction of H_2_O_2_ is inconvenient because of the high overvoltage and slow kinetics of a bare electrode [15]. Detectors, sensors and electrode syntheses depend mainly on carbon paste because of the wide voltage window, low price and low background intensity (current) [16].

The hollow polymer nanospheres allowed the dispersion of metal nanoparticles (NPs) [17]. The fabricated sensors of metal NPs have many advantages which gives such a response [18]. Palladium is a very important rare transition metal that has good catalytic activity and reported hetero-catalytic and electroanalytical behaviours [19]. The physical and chemical properties of palladium (Pd) NPs are similar to those of platinum NPs, but their price is lower. PdNPs are ideal building blocks for designing and altering nanoscale structures for specific sensing applications. They have many interesting properties such as electrocatalytic behaviour, high specific surface area, strong adsorption ability, high conductivity, improved electron transfer and reduced overpotential in electrochemical reactions [20–22]. Incorporating PdNPs in biosensors gave such high catalytic behaviour with good stability [23,24]. Electrochemical and some chemical methods have been used to incorporate PdNPs into a conducting polymer matrix [25–27]. Also, PdNPs could be dispersed in many other polymers [27,28]. H_2_O_2_ electrooxidation via PdNPs was reported [29–31]. The direct electrooxidation of the three isomers of dihydroxybenzene at lower potentials and lower detection limits was studied on PdNPs/poly1,5-diaminonaphthalene modified GC electrodes [32]. In addition, many electrochemical sensors, which were based on p1,5-DAN, were previously investigated [33–36].

Modified CPE has been applied as an electrochemical sensor for the analysis of various biologically important compounds [11,12,37–43] due to its low residual current, low cost, relative ease of electrode preparation and regeneration, and the porous surface. To the best of our knowledge, this is the first time to use PdNPs/p1,5-DAN on the surface of CPE for sensitive determination of H_2_O_2_. This approach is considered accessible and ecologically green for p1,5-DAN/CPE and PdNPs/p1,5-DAN/CPE. PdNPs/p1,5-DAN/CPE have a larger surface area, rapid mass transfer and excellent electron transfer capability compared to p1,5-DAN/CPE, imparting excellent electrocatalytic performance toward H_2_O_2_ sensing at 0.05 and −0.2 V using cyclic voltammetry (CV), and −0.12 V for square wave voltammetry (SWV) techniques.

A recent study has developed a non-enzymatic amperometric sensor by stabilizing gold (Au) NPs on a porous titanium dioxide (TiO_2_) nanotube (NTs) electrode (TiO_2_-NTs). The aggregation was prevented by entrapping AuNPs on TiO_2_ NTs. The sensor enhanced the electron transfer rate and the electrical conductivity, and generated a low detection limit of 104 nM [18]. Also, nickel oxide NPs modified multiwalled carbon nanotubes were prepared and supported on glassy carbon electrode for H_2_O_2_ sensing. The applied electrode showed good stability and reproducibility with LOD of 1.0 µM [44]. The modification of glassy carbon electrode with colloidal microcatalyst of PdNPs decorated on polyaniline coated carbon microspheres was applied for H_2_O_2_ sensing. This provides a large number of catalytic sites, high electrochemical surface area and excellent electrocatalytic activity toward H_2_O_2_ reduction with LOD of 0.7 µM [25].

## 2. Experimental

### 2.1. Instrumentations and reagents

A Voltalab Model PST006, equipped with Voltamaster4 software was used for the determination experiments [35]. The most traditionally used three-electrode cell of silver/silver chloride (Ag/AgCl) as the electrode, platinum wire (Pt) as the electrode, and CPE as the reference, auxiliary and working electrodes, respectively, was employed for all voltammetry experiments. The following chemicals were used, [1,5-Diaminonaphthalene (1,5-DAN) (97% Merck USA), graphite powder (Sigma Aldrich), NaOH (Pellets 99.8%, Merck USA), PdCl_2_ (59% Merck USA) and H_2_O_2_ (Adwic, El-Nasr pharmaceutical chemical, co, Egypt)]. Double distilled water was used to prepare all reagents. K_4_Fe(CN)_6_ (Sigma Aldrich), KCl (99.5% Sigma Aldrich), where NaCl, NaHCO_3_, Na_2_SO_4_ and CuCl_2_ (Adwic, El-Nasr pharmaceutical chemical, co, Egypt). Scanning electron microscopy (SEM) and energy dispersive X-ray spectroscopy (EDX) were provided by Nanotechnology Research Center, Kafr el-Sheikh University, Egypt, using JEOL USA, JSM IT-100.

### 2.2. Preparation of working electrode

The manual blending and mixing of graphite powder with paraffin oil ‘70 : 30 wt./wt.’ was accomplished using a mortar and pestle. Electrical contact was achieved using copper wire inside a glass tube with an internal radius of approximately 1.5 mm that enclosed the paste. After the bare electrode surface was smoothed on white paper, a smooth and shiny surface was observed. The preparation of p1,5-DAN/CPE from a well-mixed solution of 1.0 M HClO_4_ and 1.5 mM 1,5-DAN was employed using CV under the following conditions:15 cycles, potential range 0.0–0.8 V and at a scan rate of 0.02 V.s^−1^.The modified electrode was placed in a homogeneous mixture of 0.1 M HClO_4_ and 2.5 mM PdCl_2_ and analysed by CV for 25 cycles at a sweep rate ‘0.05 V.s^−1^’ between ‘−0.35 and 0.65 V' for ‘25 cycles’, as reported for PdNPs/p1,5-DAN/CPE [4]. This method has received widespread attention because it is simple, and it can ensure the high purity of sensor and the selective position of Pd on the surface [23].

## 3. Results and discussion

### 3.1. Physical characterization of the modified CPE

#### 3.1.1. Scanning electron microscopy and energy dispersive X-ray spectroscopy

**Figure 1.**
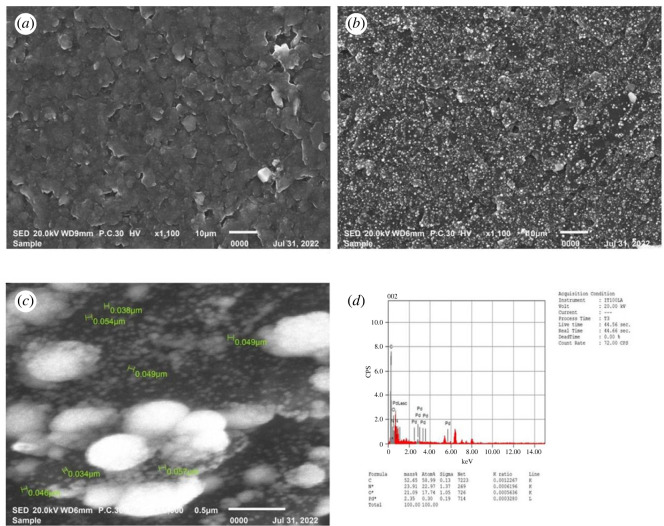
SEM images of (*a*) p1,5-DAN/CPE and (*b,c*) PdNPs/p1,5-DAN/CPE, and (*d*) EDX histogram of PdNPs/p1,5-DAN/CPE.

The response of a modified electrochemical electrode is related to its physical morphology. As shown in figure 1 *a,b*, the surface topographies of the modified electrode were analysed by SEM, which demonstrated significant differences in the surface structure of both p1,5-DAN/CPE and PdNPs/p1,5-DAN/CPE, respectively.

SEM shows that the morphologies and textures of both layers were not similar (figure 1). The structure of p1,5-DAN (figure 1 *a*) shows whitish grey, spherical, amorphous open structure porous accumulations with a large lumpy shape [36,45]. Figure 1 *b* presents PdNPs deposited on p1,5-DAN/CPE, in which Pd particles appear as white light-grey spherical spots with a mean diameter of approximately 46 nm dispersed on the modified electrode surface.

These data show that PdNPs/p1,5-DAN/CPE is affected by the incorporation of PdNPs into the p1,5-DAN structure. This structure gives a high number of effective active sites that act as supporting sites for the deposition of PdNPs [46]. The conducting polymer increased active surface area and the catalytic activity. Figure 1 *d* shows that EDX identified the presence of PdNPs in the polymeric matrix where the PdNPs were dispersed in the p1,5-DAN polymeric matrix at 2.4% for PdNPs/p1,5-DAN/CPE.

### 3.2. Electrochemical characterization of the modified CPE

#### 3.2.1. Determination of the electroactive surface area

**Figure 2.**
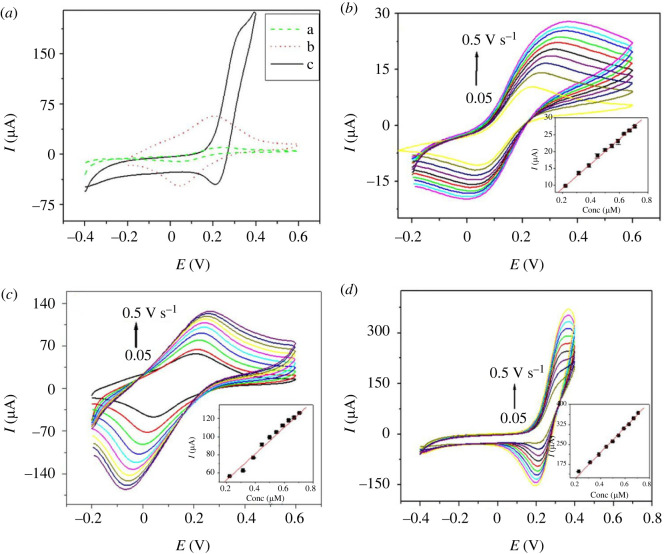
(*a*) CV traces of 1 × 10^−6^ mol.cm^−3^ K_4_Fe(CN)_6_ in 0.1 M KCl [a- CPE, b- p1,5-DAN/CPE and c- PdNPs/p1,5-DAN/CPE] at a scan rate of 0.05 V/s and CV traces for 1 × 10^−6^ mol.cm^−3^ K_4_Fe(CN)_6_ in 0.1 M KCl for (*b*) bare CPE, (*c*) p1,5-DAN/CPE and (*d*) PdNPs/p1,5-DAN/CPE at sweep rates from 0.05 to 0.5 V/s. Inset, the relationship between I_p_ and v^1/2^.

PdNPs/p1,5-DAN/CPE showed a smaller peak voltage separation ‘ΔE_p_ = 0.099 V, with a high redox intensity when tested in 1 × 10^−6^ mol.cm^−3^ [Fe(CN)_6_]^−3/−4^’ in CV, which was comparable to the bare CPE (figure 2 *a*). It exhibited better redox kinetics due to better current response, lower oxidation potential and smaller peak to peak separation and marked as quasi-reversible redox process and its redox parameters were enhanced sufficiently because of their high electric conductivity.

Equation (3.1) shows the calculation of the active surface area of the modified electrode using the Randles–Sevcik equation [47]:

3.1 $$\mathrm{Ip}=2.69\;x\;10^{5}n^{3/2}AD^{1/2}v^{1/2}[C]\;$$

where ***C*** is the K_4_Fe(CN)_6_ concentration (mol.cm^−3^), ***D*** is the diffusion coefficient (cm^2^.s^−1^), ***A*** is the surface area of the electrode (cm^2^), ***v*** is the scan rate (Vs^−1^), ***n*** is the number of transferred electrons and ***I_p_*** is the peak intensity. The square root of the scan rate and the anodic peak intensity are represented by the following equations: ***I_pa_*** = 2.006 + 36.32 v^1/2^, bare CPE; I_pa_ = 19.14 + 154.76 v^1/2^, p1,5-DAN/CPE; ***I_pa_*** = 37.31 + 458.3 v^1/2^, PdNPs/p1,5-DAN/CPE. The electroactive surface areas for PdNPs/p1,5-DAN/CPE, p1,5-DAN/CPE, and bare CPE were 0.6, 0.2, and 0.05 cm^2^, respectively. These results suggest an increase in the active surface area of the modified electrode by 12-fold compared to the bare CPE. The voltage peak difference was approximately 0.192 V where formal potential E^0^ was 0.14 V for bare CPE, as shown in figure 2 *b*. After the CPE modification with p1,5-DAN, there is a small decrease of ΔE_p_∼0.16 V with E^0^ of 0.124 V (figure 2 *c*), whereas the *Δ*E_p_ decreased to approximately 0.099 V with E^0^ of 0.25 V after the deposition of palladium nanoparticles (PdNPs), as shown in figure 2 *d* [48]. *Δ*E_p_ is inversely proportional to the electron transfer rate [49]. The bare CPE showed less electron transfer at the modified electrode surface. Accordingly, PdNPs/p1,5-DAN/CPE demonstrated the highest electron transfer rates from the lowest *Δ*E_p ∼_0.099 V (figure 2 *d*). Overall, depositing PdNPs on p1,5-DAN/CPE improved the conductivity and electrochemical properties of the catalyst.

### 3.3. Effect of the electrolyte concentration on the redox behaviour of H_2_O_2_

The strength of the basic solution influences the H_2_O_2_ decomposition rate, where the decomposition rate in a LiOH solution is 4–5 times higher than in distilled water, pH (7.0), as reported by Haines *et al.* [50]. Navarro *et al.* [51] investigated the optimal H_2_O_2_ decomposition in NaOH solutions with pH = (11.5–11.7). H_2_O_2_ oxidation in more alkaline solutions was enhanced at lower voltages, as reported by Katsounaros [52]. This study examined the effect of the electrolyte concentration using sodium hydroxide solutions.

**Figure 3.**
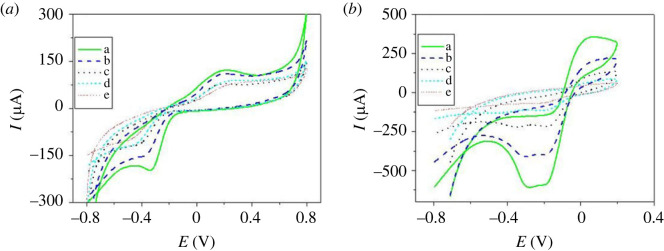
CV traces of A-24 µM H_2_O_2_ at p1,5-DAN/CPE, and B-19.5 µM H_2_O_2_ at PdNPs/p1,5-DAN/CPE in various NaOH concentrations of (*a*) 0.5, (*b*) 0.3, (*c*) 0.1, (*d*) 0.05 and (*e*) 0.02 M, sweep rate 0.05 V/s.

Figure 3 *a* presents the CV traces of 24 µM H_2_O_2_ decomposition on the p1,5-DAN/CPE electrode in various NaOH concentrations (0.02–0.5 M) with pH ranges from 12.3 to 13.7. The oxidation current, Ipa, increased from 76 to 118 µA, and the peak voltage difference (ΔE_p_) decreased from 0.751 to 0.544 V. Figure 3 *b* shows the CV trace of 19.5 µM H_2_O_2_ decomposition on the p1,5-DAN/CPE electrode in various NaOH concentrations (0.02–0.5 M). The oxidation current, Ipa, increased from 55 to 359 µA, and the peak voltage difference (ΔE_p_) decreased from 0.433 to 0.25 V. By comparing these curves, the most appropriate electrolyte for this study was 0.5 M NaOH of pH (13.7) because of the high redox current, low redox potential, and low peak-to-peak separation.

### 3.4. Effect of the pH of NaOH on the redox behaviour of H_2_O_2_

**Figure 4.**
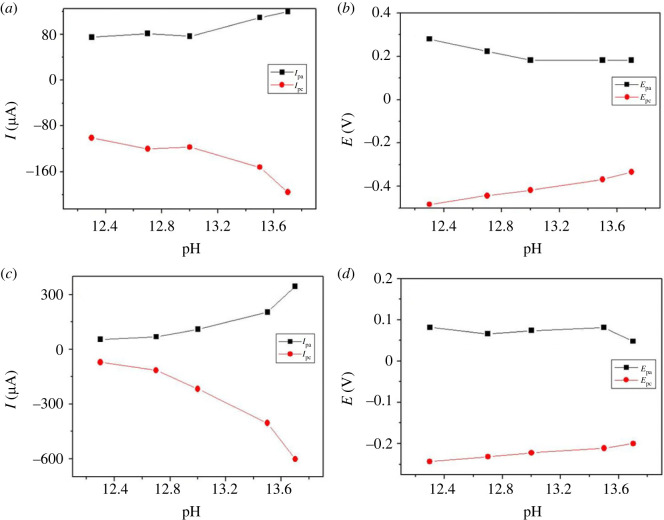
Dependence of pH on (*a*) current, (*b*) voltage of p1,5-DAN/CPE in NaOH containing 24 µM H_2_O_2_ and dependence of pH on (*c*) current, (*d*) voltage of PdNPs/p1,5-DAN/CPE in NaOH containing 19.5 µM H_2_O_2._

The pH of the supporting electrolyte influenced the value of oxidation and reduction peak voltage of H_2_O_2_, suggesting the involvement of protons in the redox reaction [53]. According to figure 4 *a*,*c*, the anodic and cathodic peak currents for redox reaction of H_2_O_2_ at the two electrodes, one of palladium and the other of palladium absence, increased with increasing pH from (12.3 to 13.7) and the maximum current observed at pH (13.7).

Figure 4 *b*,*d* showed that the anodic and cathodic peak potentials presented a dependence on pH throughout the studied range (12.3–13.7), with shifting of anodic and cathodic potential to lower energy. Therefore, pH (13.7) was determined as the optimal pH value for the electrolyte [54].

### 3.5. Effect of the scan rate

**Figure 5.**
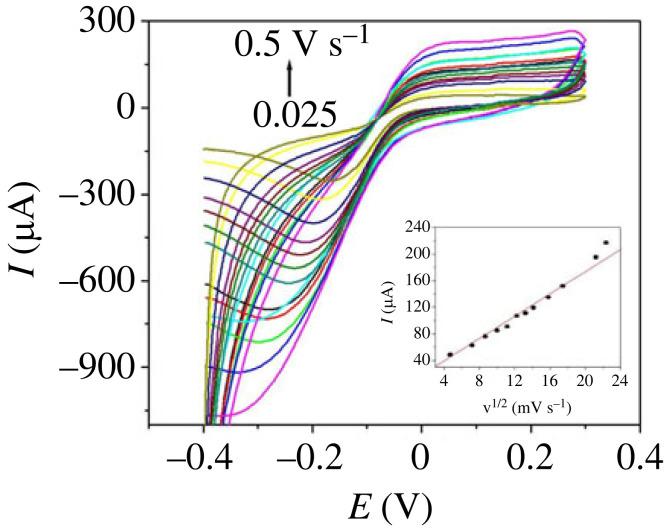
CV traces of 4.8 µM H_2_O_2_ in 0.5 M NaOH with various sweep rates from ‘0.025 to 0.5 V.s^−1^’ at PdNPs/p1,5-DAN/CPE.

PdNPs/p1,5-DAN/CPE could catalyze the H_2_O_2_ redox reaction at lower potentials with sufficiently high currents. Figure 5 shows the CV trace of 4.8 µM H_2_O_2_ with various sweep rates from 0.025–0.50 Vs^−1^ at PdNPs/p1,5-DAN/CPE.

**Figure 6.**
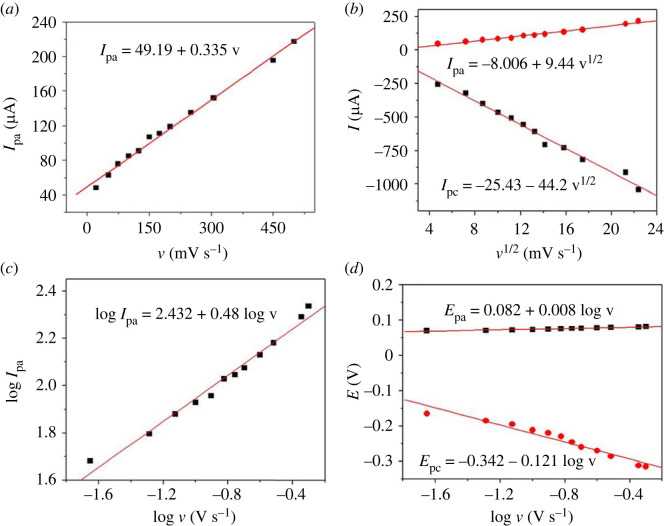
Kinetic relationships at PdNPs/p1,5-DAN/CPE for (*a*) dependence of the peak intensity *I*_pa_ and scan rate v, (*b*) plot of *I*_p_ versus v^1/2^, (*c*) plot of log *I*_pa_, versus log v, and (*d*) plot of *E*_p_ versus log v.

The redox currents increased with increasing scan rate from 0.025 to 0.1 Vs^−1^. The scan rate (v) and peak current of H_2_O_2_ showed a linear from 0.025 to 0.1 Vs^−1^(figure 6 *a*) with I_pa_ (µA) = 49.19 + 0.335 v (mVs^−1^), where I_pc_ (µA) = −193.8–2.68 v (mVs^−1^) and represented by the following equation (equation (3.2)).

3.2 $$I_{p}=\frac{n^{2}F^{2}A\Gamma \;v}{4RT}\;$$

where ***A*** is the geometric surface area (cm^2^), ***n*** is the number of transferred electrons, ***v*** is the sweep rate (Vs^−1^), ***Г*** is the surface coverage (mole/cm^2^), ***F*** is the Faraday constant (96 484 C.mol^−1^), ***T*** is the absolute room temperature (298.15 K), ***R*** is the gas constant (8.314 J.mol^−1^.K^−1^). The surface coverage for the modified electrode was 1.59 × 10^−7^ for oxidation where 1.24 × 10^−6^ mole cm^−2^ for H_2_O_2_ reduction [55].

The square root of the scan rate from 0.1–0.5 Vs^−1^ and the anodic and cathodic pulse current showed a linear relationship with increasing the scan rate. Hence, the adsorption-controlled process changed to a diffusion-controlled process [32]. Thus, the process was reported as diffusion controlled. Figure 6 *b* presents the linear XY graph between the square root of the scan rate and the H_2_O_2_ peaks intensity with I_pa_ (µA) = −8.006 + 9.44 v^1/2^ (mVs^−1^) and I_pc_ (µA) = −25.43–44.2 v^1/2^ (mVs^−1^). By applying the Randless equation (equation (3.1)), the diffusion coefficient of H_2_O_2_ at PdNPs/p1,5-DAN/CPE was 1.06 × 10^−5^ cm s^−1^ for oxidation where 4.96 × 10^−5^ cm s^−1^ for reduction [47], which was recently reported [56].

By plotting the logarithm of the scan rate versus the logarithm of the current intensity, the H_2_O_2_ redox process at PdNPs/p1,5-DAN/CPE was determined to be a diffusion-controlled process (figure 6 *c*). The slope was approximately 0.48, indicating that the reaction is diffusion-controlled [57].

A graph of the logarithm of the scan rate, log v and peak voltage (*E*_p_) for the H_2_O_2_ redox process at PdNPs/p1,5-DAN/CPE was linear at a high scan rate, as shown in figure 6 *d*. The cathodic voltages shifted to the negative direction, whereas the anodic voltages shifted to the positive direction by the gradual increase in scan rate.

3.3 $$Slope\;of\;E_{pa}=\frac{2.3RT}{(1\text{–}\alpha )nF}\;$$

3.4 $$Slope\;of\;E_{pc}=\frac{2.3RT}{\alpha nF}$$

3.5 $$\begin{array}{r} Logk_{s}=\alpha log(1-\alpha )+(1-\alpha )log\alpha -log\left(\frac{RT}{nFv}\right)-(1\text{–}\alpha )\left(\frac{\alpha F\Delta E_{p}}{2.3RT}\right), \end{array}$$

where ***α*** is the charge transfer coefficient and its value could be determined mathematically using equations (3.3), (3.4) and (3.5) and founded 0.6. The rate constant (*k*_s_) calculated using the Laviron equation was 0.22 cm.s^−1^ [55].

### 3.6. Effect of the amplitude on the redox behaviour of H_2_O_2_

The pulse amplitude is a parameter that strongly affects the peak current in square wave voltammetry affecting the sensitivity of the peak. Larger amplitudes have offered a better peak current, but as amplitudes become larger, the background current increases, the peak appears broader and shifts from its proper location it. Thus, if inappropriate amplitudes are used, it can lead to inaccurate results [58].

**Figure 7.**
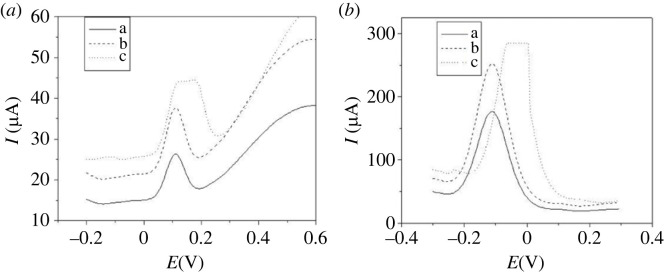
SWV of 19.5 µM H_2_O_2_ in 0.5 M NaOH with different amplitudes (a = 0.02, b = 0.05 and c = 0.07 V) at (*a*) p1,5-DAN/CPE and (*b*) PdNPs/p1,5-DAN/CPE, scan rate of 0.05 V.s^−1^, duration of 0.1 s and pulse of 0.05 V.

Figure 7 represents SWV of 19.5 µM H_2_O_2_ in 0.5 M NaOH with changing amplitude from (a = 0.02, b = 0.05 to c = 0.07 V) at (a) p1,5-DAN/CPE and (b) PdNPs/p1,5-DAN/CPE. We found that the peak current of H_2_O_2_ increased with increasing pulse amplitude from 0.02 to 0.07 V. When the pulse amplitude was higher than 0.05 V, the oxidation peak was wider and broader [33].

### 3.7. Electrochemical behaviour of H_2_O_2_

#### 3.7.1. Cyclic voltammetry behaviour

**Figure 8.**
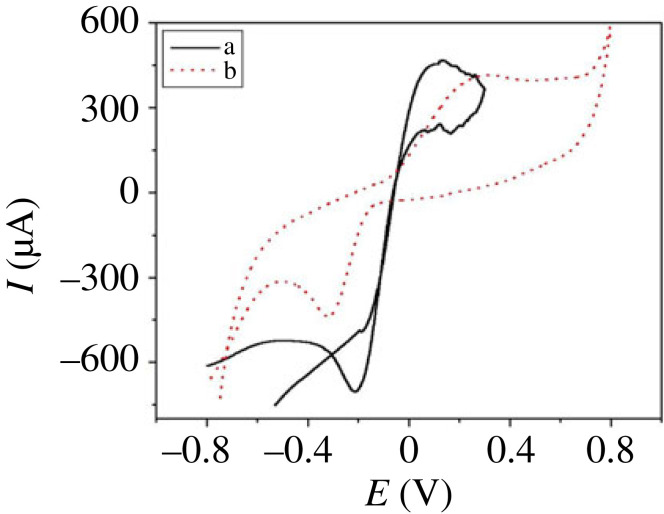
CV traces of ‘78.3 µM H_2_O_2_’ in ‘0.5 M NaOH’ recorded at (*a*) PdNPs/p1,5-DAN/CPE and (*b*) p1,5-DAN/CPE, sweep rate 0.05 V/s.

Figure 8 shows the CV response of H_2_O_2_, representing a typical CV trace of 78.3 µM H_2_O_2_ in 0.5 M NaOH at (a) PdNPs/p1,5-DAN/CPE and (b) p1,5-DAN/CPE, at a sweep rate of 0.05 Vs^−1^. By modification of CPE by only p1,5-DAN/CPE, an anodic peak appeared at *E*_pa_ = 0.222 V, *I*_pa_ = 415 µA and a cathodic peak of *E*_pc_ = −0.322 V, *I*_pc_ = 420 µA. In the case of adding a second layer of palladium nanoparticles onto the polymer film, a well-characterized and enhanced anodic peak was observed with *E*_pa_ = 0.05 V, *I*_pa_ = 466 µA, and a cathodic peak of *E*_pc_ = −0.2 V, *I*_pc_ = −705 µA. The peak-to-peak separation (ΔE) and formal potential were 0.25 and 0.125 V for PdNPs/p1,5-DAN/CPE compared to 0.544 and 0.272 V for p1,5-DAN/CPE. The PdNPs impart very high electrocatalytic activity for H_2_O_2_ catalytic oxidation and reduction at low redox potentials compared to p1,5-DAN/CPE. The PdNPs/p1,5-DAN/CPE improved the CV sensitivity for H_2_O_2_ because of the ease of electron transfer in catalytic reactions [59].

#### 3.7.2. Behaviour of square wave voltammetry ‘SWV’

The recognition of the background current and low detection limit were examined by SWV [33]. This technique was employed for H_2_O_2_ oxidation at PdNPs/p1,5-DAN/CPE because of its low contribution to the background current and high current sensitivity.

**Figure 9.**
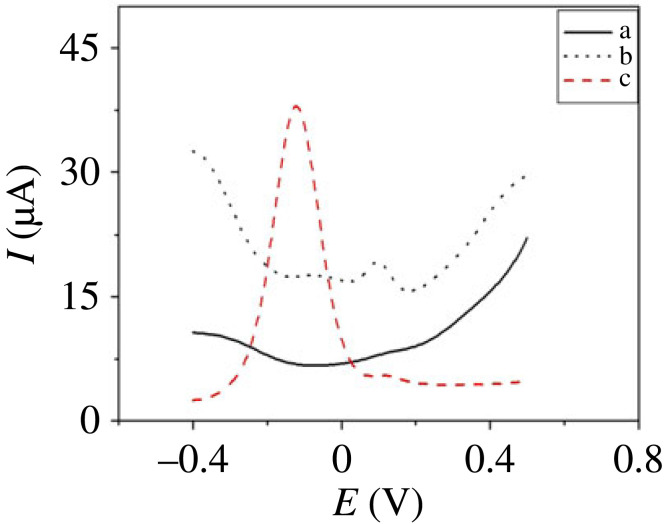
SWV of (*a*) absence of 3.2 µM H_2_O_2_ in 0.5 M NaOH at PdNPs/p1,5-DAN/CPE, (*b*) 3.2 µM H_2_O_2_ in 0.5 M NaOH at p1,5-DAN/CPE, and (*c*) at PdNPs/p1,5-DAN/CPE, at a scan rate of 0.05 V.s^−1^, duration of 0.1 s, amplitude of 0.05 V and pulse of 0.05 V.

Figure 9 presents the SWV trace of PdNPs/p1,5-DAN/CPE and p1,5-DAN/CPE in 0.5 M NaOH containing 3.2 µM H_2_O_2_, the oxidation peaks with anodic currents were at −0.12 and 0.115 V for 40.6 and 19.5 µA, respectively. From the anodic peak potential and current, PdNPs/p1,5-DAN/CPE can improve H_2_O_2_ detection corresponding to the CV results.

### 3.8. Determination of H_2_O_2_

#### 3.8.1. Cyclic voltammetry determination

**Figure 10.**
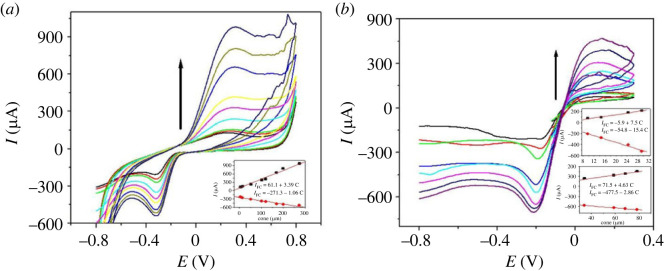
CV traces of H_2_O_2_ in 0.5 M NaOH at (*a*) p1,5-DAN/CPE from 4.9 to 274 µM and (*b*) PdNPs/p1,5-DAN/CPE from 9.7 to 78.3 µM, scan rate of 0.05 V.s^−1^.

CV demonstrated the best determination of H_2_O_2_ at PdNPs/p1,5-DAN/CPE. Figure 10 *a* shows the CV traces for various H_2_O_2_ concentrations in 0.5 M NaOH at p1,5-DAN/CPE, and its inset shows a linear relationship for the redox concentrations and their currents. The linear regression equation for anodic oxidation was I_pa_ (µA) = 61.1 + 3.39 C (µM) with a correlation coefficient of 0.99, where I_pc_ (µA) = −271.3−1.06 C (µM) for cathodic reduction. The lower detection (LOD) and lower quantification (LOQ) limits for H_2_O_2_ oxidation by CV were 0.3 and 0.99 µM and for its reduction were 0.4 and 1.32 µM.

Figure 10 *b* presents the CV traces for the H_2_O_2_ concentrations in 0.5 M NaOH at PdNPs/p1,5-DAN/CPE. The inset showed the linear relationship between the redox concentrations and their currents. The linear regression equation for anodic oxidation was *I*_pa_ (µA) = −5.9 + 7.5 C (µM) with a correlation coefficient of 0.99 where *I*_pc_ (µA) = −54.8−15.4 C (µM) for cathodic reduction. The limit of detection (LOD) and limit of quantification (LOQ) for H_2_O_2_ oxidation by CV were 5.0 and 16.6 nM, respectively, whereas they were 4.0 and 13.3 nM for its reduction, respectively. The LOD and LOQ values were calculated using equations (3.6) and (3.7).

3.6 $$LOD=\frac{3\sigma }{m}$$

and

3.7 $$LOQ=\frac{10\sigma }{m}\;$$

where σ is the blank standard deviation and ***m*** is the linear's slope [16].

These results suggest that the PdNPs/p1,5-DAN/CPE can effectively mediate electron transfer between the electrode and H_2_O_2_, showing clear catalytic activity toward electrochemical redox determination. This approach can be applied to the determination of a lower H_2_O_2_ concentration.

The electrochemical H_2_O_2_ reduction mechanism was reported previously [60,61] as equations (3.8)–(3.10).

3.8 $$H_{2}O_{2}+\bar{e}⇌{\mathrm{OH}}_{\mathrm{ads}}\;+{\mathrm{OH}}^{-}\;$$

3.9 $${\mathrm{OH}}_{\mathrm{ads}}+\bar{e}⇌{\mathrm{OH}}^{-}$$

3.10 $$\mathrm{and}\;2{\mathrm{OH}}^{-}+2H^{+}⇌2H_{2}O$$

The polymer film (1,5-DAN) has many imino groups (─NH) which have constructed hydrogen bonds leading to a decrease in the hydroxyl bond energy through an O─H**^….^** NH bond [32]. Moreover, PdNPs have created more O─H**^….^** PdNPs bonds which helped in the electron transfer [62].

This electron transfer has gained by adsorbed H_2_O_2_ on the electrocatalyst surface which produces (OH)_ads_ and OH^─^ as in equation (3.8). Subsequently, another electron is received by (OH)_ads_ resulting in H_2_O formation. In all processes, the reaction rate is influenced primarily by two factors : (1) H_2_O_2_ adsorption at the electrocatalyst surface and (2) electron transfer from the electrocatalyst to the (OH)_ads_. Thus, the electrocatalyst must enhance the adsorption and electron transfer to establish the electrocatalytic reduction successfully [63].

The sensitivity determination of p1,5-DAN/CPE and PdNPs/p1,5-DAN/CPE were 12 500 µAmM^−1^ cm^−2^ and 16 950 µAmM^−1^ cm^−2^, respectively, according to equation (3.11) [64].

3.11 $$\mathrm{Sensitivity}=\frac{\left[m\right]}{\left[A\right]}\;$$

where ***m*** is the linear slope and ***A*** the electrode active area.

#### 3.8.2. Square wave voltammetry of H_2_O_2_

**Figure 11.**
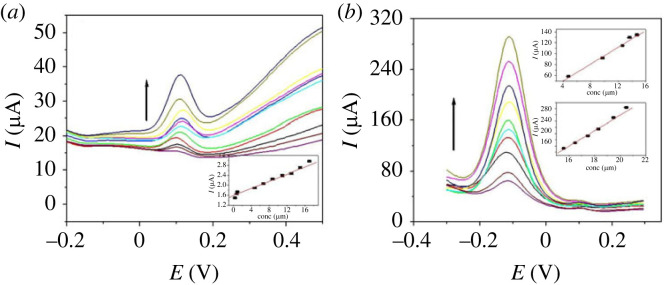
SWV of H_2_O_2_ in 0.5 M NaOH (*a*) at p1,5-DAN/CPE from 0.45 to 19 µM and (*b*) at PdNPs/p1,5-DAN/CPE from 4.8 to 20.5 µM, scan rate of 0.05 V.s^−1^, duration of 0.1 s amplitude of 0.05 V and pulse of 0.05 V.

Figure 11 *a* shows the SWV for p1,5-DAN/CPE in different concentrations of H_2_O_2_ from 0.45 to 19 µM. Figure 11 *b* represents the SWV of PdNPs/p1,5-DAN/CPE in different concentrations of H_2_O_2_ from 4.8 to 20.5 µM. The inset of figure 11 *a*,*b* shows linear graphs of the peak versus H_2_O_2_ concentrations. The p1,5-DAN/CPE's coefficient (*R* ^2^) was 0.99 with *I*_pa_ (µA) = 15.5 + 0.77 C of H_2_O_2_, where 0.99 with *I*_pa_ (µA) = −276.3 + 27.07 C for PdNPs/p1,5-DAN/CPE. The LOD, LOQ and sensitivity were 0.038 µM, 0.126 µM and 3850 µAmM^−1^ cm^−2^, respectively, at p1,5-DAN/CPE. However, the values for PdNPs/p1,5-DAN/CPE were 1.0 nM, 3.32 nM and 45 000 µAmM^−1^ cm^−2^, respectively. The Pd in the hollow polymer matrix was responsible for the excellent performance, because it increases the surface area and provides many active sites for the catalytic reactions that in turn improves the sensitivity, selectivity and conductivity of the sensor [32].

Table 1 lists the LODs for H_2_O_2_ determination at both p1,5-DAN/CPE and PdNPs/p1,5-DAN/CPE compared with previously investigated modified electrodes. From table 1, in comparison with previously reported CPE, PdNPs/p1,5-DAN/CPE could determine H_2_O_2_ at a good lower concentration limit with low redox potentials. Also, the electrochemical determination of H_2_O_2_ at PdNPs/poly 1,5-DAN/CPE has not been previously performed, ensuring the novelty of our work.

**Table 1.** Electrochemical determination of H_2_O_2_ at different modified electrodes. Abbreviations: (AP-Ni-MOF Ni^+2^-based metal-organic framework) (PNMA [SDS]the polymer of N-methylaniline which was synthesized in the presence of sodium dodecyl sulfate) (SCE saturated calomel electrode) (PVA polyvinyl alcohol) ((PEDOT:PSS poly 3,4-ethylenedioxythiophene: polystyrene sulfonate) (HRP horseradish peroxidase) (PGN porous graphene) (TiO_2_ titanium dioxide) (Ag@PPy-C silver decorated polypyrrole-carbon black) (CS/GOP crosslinked chitosan and polyaniline grafted grapheme oxide composite) (NiO, nickel oxide nanoparticles- CNT, modified multiwalled carbon nanotubes- PEI, polyethyleneimine) [(Au(pc) polycrystalline gold electrode] (sub-CYST sub-monolayer coverage of cysteine).

electrode	electrocatalytic mediator	electrocatalytic effect (mV)	LOD (µM)	LDR (µM)	references
**CPE**	**p-1,5-DAN**	**E_pa_ = 222**	**0.3 (CV)**	**0.6–283**	**this work**
		**E_pc_ =** −**322**	**0.4 (CV)**	**0.6–283**	
		**E_pa_ = 115**	**0.038 (SWV)**	**0.2–200**	
**CPE**	**PdNPs/p-1,5-DAN**	**E_pa_ = 50**	**0.005 (CV)**	**0.2–300**	**this work**
		**E_pc_ =** −**200**	**0.004 (CV)**	**0.2–300**	
		**E_pa_ =** −**120**	**0.001(SWV)**	**0.1–250**	
CPE	AP-Ni-MOF	E_pc_ = −250	0.9	4–60 000	[65]
CPE	PNMA(SDS)/Co	E_pa_ = 400	CV =18	30–12 000	[66]
			DPV = 3	5–48	
			SWV = 0.9	1–12	
GCE	PtPd/MWCNTs	E_pa_ = 250	1.2	2.5–125	[67]
GCE	gold nanoparticle-silica sol-gel	E_pc_ = −500 (versus SCE)	0.003	2.5–45	[68]
GCE	PVA/MWCNTs/PtNPs	E_pc_ = 0	0.7	2–3,800	[69]
GCE	AgNPs/MWCNT/rGO/	E_pc_ = −350	0.9	0.1–100	[70]
ITO	polyaniline grafted MWNT	E_pc_ = −300 (versus SCE)	0.001	0.01–0.2	[71]
GCE	Prussian blue	E_pa_ = 50	0.01	0.01–10 000	[72]
GC rotating electrode	cobalt oxide nanoparticles	E_pa_ = 750	0.0004	0.004–0.08	[73]
Pt	PVA/AgNPs	E_pc_ = −553 (versus SCE)	1.0	1.2–1,000	[74]
GCE	MnO_2_ Nanosheets	E_pc_ = −600	0.0005	0.025–454	[75]
GCE	Cs micelle/PEDOT: PSS/HRP/Nafion	E_pc_ = −238	0.00003	0.0001–10	[76]
GCE	HRP/PGN	E_pc_ = −700 (versus SCE)	0.00002	2.77–835	[77]
TiO_2_ NPs	AuNPs	E_pc_ = −350	0.104	1–198	[18]
GCE	Ag@PPy-C/TiO_2_ NCs	E_pa_ = 250	0.23	5–57	[78]
GCE	CS/GOP	E_pc_ = −470	17.3	0.5–200	[63]
GCE	CNT-PEI@NiO	E_pa_ = 600	1.0	4–800	[44]
Au(pc)	sub-CYST	E_pa_ = −100	0.8	1–3,000	[79]

### 3.9. Real samples analysis

This study examined H_2_O_2_ in various spiked water sources (canned, underground, tap water) using the very good response electrode PdNPs/p1,5-DAN/CPE. No permissions were required prior to conducting field studies. A 10 ml cell of a real sample of 0.5 M NaOH was used for the spiked sample 0.1 mM H_2_O_2_ / 0.5 M NaOH using the standard addition method, while SWV was applied using the PdNPs/p1,5-DAN/CPE. Table 2 lists the average recoveries of all specimens repeated four times. Data analysis showed that the fabricated modified electrode could determine different H_2_O_2_ concentrations in real samples. The good recovery and response, easy preparation, and low cost give a chance for the PdNPs/p1,5-DAN/CPE for its use in industry as a fast detector of H_2_O_2_.

**Table 2.** Determination of H_2_O_2_ in real water samples at PdNPs/p1,5-DAN/CPE.

	samples	added (µM)	found recovery (µM)	(%)	^n^RSD (%)
**tap**	*1*	4.62	4.53	98.1	0.46
	*2*	4.66	4.66	100	0.19
	*3*	4.68	4.5	96.2	0.31
**underground**	*1*	4.8	4.62	96.3	0.45
	*2*	2.38	2.51	105.5	0.85
	*3*	3.63	3.43	94.4	1.09
**canned**	*1*	4.51	4.18	92.6	0.61
	*2*	2.15	2.15	100	0.97
	*3*	4.49	4.23	94.1	0.51

^n^ RSD% calculated from four measurements.

### 3.10. Interference study

The selectivity of PdNPs/p1,5-DAN/CPE toward H_2_O_2_ determination was examined by determining the effects of potentially interfering species, including 4.97 µM urea, 4.97 µM ascorbic acid, 24.8 mM Na^+^, 24.8 mM Cl^−^, 24.8 mM K^+^, 24.8 mM ${\mathrm{HCO}}_{3}^{-}$ , 9.9 mM ${\mathrm{SO}}_{4}^{-2}$ , 4.9 mM Cu^+2^, on PdNPs/p1,5-DAN/CPE containing 4.97 µM H_2_O_2_. Table 3 confirms the absence of interference with H_2_O_2_ peak current, only a signal change ≤5%. Thus PdNPs/p1,5-DAN/CPE has good selectivity toward H_2_O_2_ determination.

**Table 3.** Interfering species effects on H_2_O_2_ determination.

sample number	samples	% signal
**1**	urea	0.94
**2**	ascorbic acid	0.47
**3**	Na^+^	2.35
**4**	Cl^−^	1.1
**5**	K^+^	1.72
**6**	HCO_3_^−^	3.4
**7**	SO_4_^−2^	2.5
**8**	Cu^+2^	1.72

### 3.11. Stability and reproducibility of the PdNPs/p1,5-DAN/CPE

**Figure 12.**
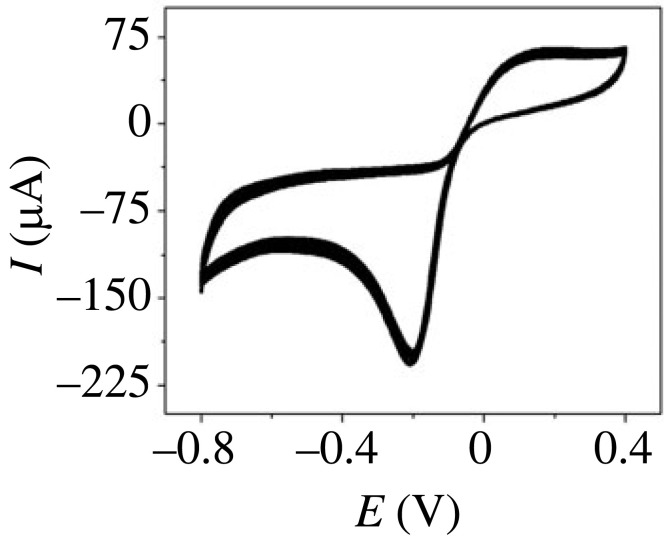
CV trace of 4.8 µM H_2_O_2_ in 0.5 M NaOH at PdNPs/p1,5-DAN/CPE for 30 cycles, scan rate 0.025 V.s^−1^.

The stability, reversibility and reproducibility of PdNPs/p1,5-DAN/CPE are important factors for practical applications [80]. Figure 12 presents the CV traces of 4.8 µM H_2_O_2_ in 0.5 M NaOH for 30 successive cycles. The antifouling surface of the electrode was indicated by stable redox voltage and peak intensity. In addition to the intra-day precisions, the convenient tools for checking the electrode confirmed its reproducibility. The precision of the assay was tested by examining the sensor behaviour in one determined solution four times. The good precision of the assay was highlighted by the low RSD value of 0.62%. Ten different PdNPs/p1,5-DAN/CPE were prepared for the evaluation of reproducibility, five for intra-day and five for inter-day measurement. Each prepared electrode was used to measure the current intensity response of 4.8 µM H_2_O_2_ in 0.5 M NaOH. For the five intra-day fabricated electrodes the RSD of the five current intensities was 0.62% and 0.85% for the five inter-day prepared electrodes.

## 4. Conclusion

In this study, the double character of DAN (a hollow film) and PdNPs improved the electrochemical redox response toward H_2_O_2_ via increasing the effective surface area and electron transfer. The PdNPs/p1,5-DAN/CPE has become a promising sensor to determine H_2_O_2_ in real samples, environmental samples, and in enzymeless catalyzed reactions due to its advantages of high sensitivity, reproducibility, good stability and anti-interference ability against many interferents. The assay was simple and inexpensive for H_2_O_2_ determination with a lower detection limit and good precision.

## Data Availability

Source data were submitted to dryad through linkage https://doi.org/10.5061/dryad.3tx95x6nz [81].
